# Canned Provisions

**Published:** 1883-12

**Authors:** 


### CANNED PROVISIONS.

The use of canned goods is now so universal that any information concerning their healthfulness is of interest. We therefore, condense the following from the *Medical Times and Gazette*, October 27th, 1883:

Tinned meats, soups, vegetables, and more especially fruits, are all, without exception, contaminated by metals; such is the conclusion of investigation. In 1878, Mr. Albert E. Menke communicated to the *Chemical News* results of analyses of a tin of lobster, one of apples, and another of pineapple; the latter contained tin dissolved in the juice equal to 1.3 grain per pound, the lobster and apples a much smaller quantity. Mr. Hehner, in 1880, communicated to the *Analyst* the results of an investigation of the subject. He found tin in tinned French asparagus, American asparagus, peas, tomatoes, peaches, pineapples, white cherries, red cherries, marmalade, corned beef (five different brands), ox-cheek, ox-tongue (three kinds), collared head, tripe, oysters, sardines preserved in oil, salmon, lobsters, shrimps, curried fowl (two kinds), boiled rabbit, boiled mutton, roast chicken, roast turkey, soup, and in three brands of condensed milk. The amount of tin found does not appear large—*e. g.*, in the milk one-tenth of a grain per pound, in one of the soups half a grain per pound, and in a pound tin of preserved oysters seven-tenths of a grain per pound. On a later research, Mr. Wynter Blyth has found larger quantities. In a recent report detailing the examination of twenty-three samples of tinned apricots, tomatoes, pineapples, and cranberries, the amounts found calculated as stannous hydrate range from 1.9 grain to 14.3 grains per pound, the mean amount being 5.2 grains. The juice and fruit in some instances had a metallic taste. Several of the tins showed signs of corrosion.

The older school of toxicologists, as represented by Orfila, considered pure tin vessels innocuous; if accidents occurred, they were ascribed to admixture of lead in the alloy, or to arsenic. The arsenic theory ceased to be held when it was found that arsenic was present in so small a quantity that an adult would have to spend more than forty years drinking and eating from tin vessels before he imbibed a poisonous dose; even the explanation of lead so often accompanying the tin has not of late been considered sufficient, but the question is of some moment whether tin in itself present in a soluble form contaminating food, may not act injuriously. All know the toxic action of the chloride of tin on the one hand, and the inactivity of stannic oxide on the other; it is evident that in tinned foods we have to do with neither, but with some form of stannous hydrate. The little that is known of the action of stannous hydrate may be briefly given.

Doses of .174 gramme per kilogramme of body weight cause in guinea-pigs death with signs of intestinal irritation; but with doses smaller than .17 to .2 gramme the effects are uncertain, and the animals generally recover. Hence, supposing a man to be affected in the same proportion, he would have to take from three to four drachms, or consume at a meal ten pounds of the most contaminated of Mr. Wynter Blyth's tinned fruits.

But it is not a question of immediate mortality, it is an inquiry as to the action of repeated small doses continued for a long time. In summer, families who go yachting, fishing, or traveling in remote parts, often carry a supply of "tinned" provisions, and must take in the aggregate, physiologically active doses of stannous hydrate, possibly producing slight dyspepsia or intestinal irritation. From time to time, indeed, serious symptoms are witnessed after eating tinned meats; but the exact cause of such illness has never been in any thorough way investigated. It must also be remembered that certain sugars now in the market contain tin in the proportion of about half a grain to the pound—no very great quantity in itself, but the small fractions of the metal found in this and that article of food in daily use may mount up until an active dose is taken. Physicians will do well to inquire closely into the diet of their patients suffering from obscure gastric affections.

